# The association between domestic violence exposure and cyberbullying behavior among secondary school students

**DOI:** 10.3389/fpsyt.2023.1302477

**Published:** 2023-12-22

**Authors:** Di Chen, Boyang Xu, Jing Chen

**Affiliations:** ^1^School of Criminology, People's Public Security University of China, Beijing, China; ^2^School of Criminal Justice, China University of Political Science and Law, Beijing, China; ^3^College of Humanities & Social Sciences, Huazhong Agricultural University, Wuhan, China

**Keywords:** cyberbullying behavior, domestic violence exposure, depression, self-control, secondary school students

## Abstract

**Introduction:**

Cyberbullying could have a severe and long-term impact on the physical and mental health of secondary school students because of its characteristics of being hidden, repetitive, and exceeding the limitations of time and space, thus attracting widespread attention. Among the many environmental factors, family was the immediate environment where secondary school students live. Violent behaviors such as aggression displayed by other subjects in the family environment could trigger aggressive behaviors among secondary school students, and the indirectness of the online environment intensifies this tendency.

**Methods:**

This study used the Cyberbullying Scale, the Domestic Violence Exposure Scale, the Depression Scale, and the Brief Self-Control Scale to conduct a questionnaire survey of 10,273 secondary school students in 12 secondary schools from Liaoning, Zhejiang, and Henan provinces in China to explore the relationship and internal mechanisms between domestic violence exposure and cyberbullying behavior among secondary school students.

**Results:**

This study found that (1) domestic violence exposure has a significant positive associated with cyberbullying behavior among secondary school students; (2) the mediating effect of depression partially exists between domestic violence exposure and cyberbullying behavior among secondary school students; (3). self-control alleviated the effects of domestic violence exposure on cyberbullying among secondary school students but intensified the effects of depression on cyberbullying among secondary school students.

**Discussion:**

The results suggest that while focusing on the association of domestic violence exposure with cyberbullying among secondary school students, it is also necessary to pay attention to the mediating effect of depression and the mitigating and intensifying mechanisms of self-control, resulting in a notable weakening effect on cyberbullying among secondary school students.

## 1 Introduction

The *51st Statistical Report on China’s Internet Development* showed that as of December 2022, the number of Internet users in China reached 1.067 billion, and youths aged 10–19 accounted for 14.3% of Internet users (1). A UNICEF online survey showed that 24% of students aged 10–18 in China were subjected to cyberbullying, which has become a social and public health problem that requires great attention (2). Cyberbullying includes both the perpetrator and the victim, and this study focused on preventing and reducing cyberbullying from the perspective of the perpetrator. Cyberbullying refers to aggressive behavior by an individual or group of individuals who repeatedly convey hostile or offensive messages through electronic or digital media to cause harm or discomfort to others (3). Adolescents were socially connected to others through communication tools, and these tools became a new vehicle for bullying behavior.

Cyberbullying happens mainly through verbal and relational bullying, including harassment, defamation, impersonation, ostracization, and online spectating (4). The new existing forms made bullying ubiquitous, occurring at a high rate during adolescents’ school, home, or travel. Bullies hidden behind communication tools could not perceive the direct reaction of the bullied, reducing the empathy generated by seeing the pain of the bullied and weakening the inhibitory effect on the infliction of pain, resulting in a greater tendency for bullying to occur. Studies showed that compared to physical bullying, cyberbullying could cause more severe and longer-lasting psychological trauma for the victim (5). Therefore, an in-depth discussion of the influencing factors of cyberbullying behavior was vital for preventing and reducing such deviant behaviors and enhancing students’ psychological health.

Previous studies often had problems with small samples and limited quantity. The uniqueness of this study lay in the following: first, as a vast country, China had significant differences in the natural geographic environment, historical legacy, religious beliefs, regional dialects, and economic development of different regions, resulting in significant differences in cultural values, customs, and behaviors across different regions (6). The sample of this study covered three different provinces, namely, the northern part of the country (Liaoning Province), the central plains of the country (Henan Province), and the southern part of the country (Zhejiang Province), which ensured the geographic heterogeneity of the sample; second, the sample is scientifically selected by proportion for the number of secondary schools and students in each province, and the sample size was greater than 10,000, which made the research results more robust and credible; Finally, using integrated combinations of different variables could innovatively improve the effectiveness for explaining the models. The result could support and inspire more similar empirical studies in this field.

Violence exposure (VE) was the individual’s exposure to information related to violence as a stimulus in their lives (7). Ecological system theory (EST) addressed that several behavioral systems would influence adolescent development. The family was a microsystem closely related to the individual (8). Scholars argued that negative factors in the family were significantly associated with adolescent aggressive behavior (9), especially domestic violence exposure (10–12). For secondary school students, the main pathways of exposure to domestic violence included witnessing and experiencing domestic violence firsthand. Witnessing violence was when an individual saw violence being committed or suffered by a family member (e.g., parental physical conflict), and experiencing violence was when an individual is personally victimized by violence (e.g., physical abuse) (13).

According to social learning theory, adolescents learn parental or adult behaviors through observation and imitation (14). Adolescents who had witnessed or experienced domestic violence tended to view violence as an acceptable approach to resolving interpersonal conflicts and internalize this as a stable personality trait, increasing the likelihood that they would engage in bullying behaviors in the future (15, 16). A meta-analytic study showed that exposure to domestic violence significantly impacted both emotional and behavioral problems in adolescents (17), and witnessing inter-parental violence was more significant than witnessing other forms of destructive conflict (18, 19).

In addition, a family-tracking study found that among multiple interpersonal violence exposures, aggression between parents and parents toward their children significantly increased adolescents’ internalizing (e.g., depression) and externalizing (e.g., bullying behavior) problems (20).

Based on this, this study proposed H1:

H1: Domestic violence exposure is significantly associated with cyberbullying behavior among secondary school students.

Depression was a negative emotional response. According to the general strain theory, a bad external environment can trigger psychological stress in an individual, which in turn could cause the individual to restore a balanced psychological state by committing cyberbullying behaviors (19). A domestic violence background was a potential risk factor for mental health problems such as depression in adolescents (21). Growing up in a domestic violence environment could cause adolescents to subjectively develop negative emotions such as a sense of pain and despair. The repeated stimulation of violent cues dragged adolescents under a tremendous psychological burden, which would develop and accumulate a large amount of depression (22, 23) and may initiate bullying behavior during online social interactions to release or alleviate psychological stress. Empirical studies have shown that adolescents exposed to violent cues have significantly higher levels of depression than normal individuals (24), and those who reported high levels of depressive symptoms tended to have difficulties in suppressing impulsive behaviors and obtaining effective emotion adjustment strategies (25). Thus, depressive symptoms have a significant effect on cyberbullying behavior (26, 27). Accordingly, this study proposes H2.

H2: The mediating effect of depression exists between domestic violence exposure and cyberbullying behavior among secondary school students.

Self-control signified an individual’s ability to consciously control impulsive behavior to resist satisfying immediate needs and desires to obtain long-term benefits (28). According to the dual-system model, both the impulsive and reflective systems influence an individual’s behavior in response to stimuli when facing external lures (29). Specifically, individuals with high levels of aggression would generate aggressive impulses due to cognitive preferences when exposed to violent stimuli.

However, external personal criteria (reflective system) would limit the aggressive impulses, and self-control would then play a corresponding role in the conflict process of the dual systems. Individuals with high self-control were more likely to be influenced by external personal criteria, self-regulating negative emotions, reinforcing delayed gratification, and trying to avoid behaviors detrimental to their self-interest. Individuals with low self-control were more affected by internal impulses, experiencing cognitive biases, impaired emotion regulation, and accumulated negative emotions, which in turn triggered aggressive behaviors (30). Therefore, due to the “reflective” role of personal criteria, adolescents with a high level of self-control tended to proactively inhibit the triggering process of cyberbullying behavior by violent stimuli and vice versa. This study proposes H3:

H3a: Self-control moderates the association of domestic violence exposure and cyberbullying behavior among secondary school students.

H3b: Self-control moderates the association of domestic violence exposure and depression among secondary school students.

H3c: Self-control moderates the association of depression and cyberbullying behavior among secondary school students.

**Figure 1:**
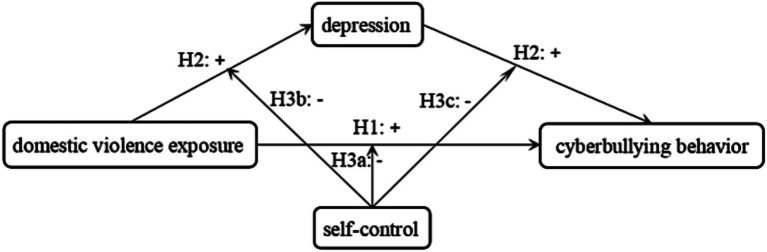
Hypothesis map.

All hypothesized routes for this study can be found in Figure 1.

## 2 Method

### 2.1 Participants

In this study, screening criteria were established based on the level of economic development, geographic location, demographic characteristics, the number of secondary schools, and the number of enrolled students, respectively. Liaoning, Henan, and Zhejiang Provinces were selected as the sampling sites for secondary school studenrs, ranging from middle school to high school, i.e., grades 7 to 12. Based on the principle of purposive stratified cluster sampling and the proportion of the number of secondary schools and students in the three provinces (approximately 1:2:3), two secondary schools in Liaoning Province, four secondary schools in Zhejiang Province, and six secondary schools in Henan Province were ultimately selected as the sampling schools for this study. The questionnaire for this study was approved by the Ethics Committee of the People’s Public Security University of China, and a confidentiality and ethics statement was provided at the top of the questionnaire with the notification informing that submitted questionnaires indicated the voluntary consent of the participants.

The questionnaires were distributed through class-based self-organized social media groups via online links (URLs) to “Wen Juan Xing (lit. Questionnaire Star, www.wjx.cn)” or in paper form during free study hours at school under the researcher’s on-site guidance. As a result, a total of 10,640 questionnaires were collected from 12 secondary schools, and after excluding invalid questionnaires, 10,273 valid questionnaires were retained, with a completion rate of 96.6%. Of the valid samples, 1883 were from Liaoning Province, 5,125 from Henan Province, and 3,265 from Zhejiang Province.

### 2.2 Measures

#### 2.2.1 Cyberbullying scale

The Cyberbullying Scale was from the Cyberbullying Subscale in the Bullying Scale for secondary school Students developed by Olweus (31), edited by Ji, YanTing (32). The questionnaire consisted of five items. It includes five types of cyberbullying behaviors, including text intimidation, public texting, kicking out of group chats, spreading rumors, and verbally abusing others online. The scale was a 5-point scale (1 = did not occur, 2 = occurred once or twice, 3 = two or three times a month, 4 = once a week, 5 = several times a week), and a higher score indicated that an individual was committing cyberbullying to a more severe degree. The Cronbach’s α coefficient for this scale in this study was 0.957.

#### 2.2.2 The domestic violence exposure scale

The Domestic Violence Exposure Scale (DVES) from the Multiple Forms of Violence Scale (MFVS) developed by Ho et al. (7) was used, which was divided into two dimensions: exposure to witnessing violence and exposure to experiencing violence. The questionnaire consists of 16 items and is based on a five-point scale (1 = never, 2 = once, 3 = several times, 4 = multiple times, and 5 = every day), with higher scores indicating that the individual has been exposed to violent situations to a greater extent in their lives. The Cronbach’s α coefficient for this scale in this study was 0.930.

#### 2.2.3 The depression scale

The *Depression Scale* (DS) developed by Radloff and revised by Chen, Zhiyan et al. was used in the Chinese version of Radloff’s *The Center for Epidemiological Studies Depression Scale (CES-D)* (33). The questionnaire consists of eight items and is scored on a five-point Likert scale (from 1 = strongly disagree to 5 = strongly agree), with higher scores indicating higher levels of depression in individuals. The Cronbach’s *α* coefficient for this scale in this study was 0.954.

#### 2.2.4 The brief self-control scale

The *Brief Self-Control Scale* (*BSCS*) (34) developed by Morean (35) and revised by Luo, Tao et al. was used, which consists of 7 items and includes two dimensions: Self-Discipline and Impulse Control. The scale was scored on a 5-point Likert scale (1 = not at all to 5 = fully match), with items 2, 4, 6, and 7 being reverse-scored questions. The total sum score is calculated for all items, with higher scores indicating a higher level of individual self-control. The Cronbach’s *α* coefficient for this scale in this study was 0.803.

### 2.3 Data analysis

Reliability tests, common method bias tests, descriptive statistics, and Pearson’s correlation analyses were performed using the SPSS 26.0 software. Mediation and moderation were examined using Model 59 in the Process 3.3 plug-in of the SPSS 26.0 software and the Johnson-Neyman method (J-N method).

### 2.4 Common method bias test

In this study, common method bias was procedurally controlled by taking measures such as anonymous surveys and reverse scoring of some items. The collected data were examined for common method bias using the Harman one-factor test, and the results of the unrotated exploratory factor analysis extracted a total of seven factors with an eigenroot greater than 1, with a maximum factor variance explained rate of 27.89% (less than 40%) (36). Therefore, the approach of the questionnaire used in this study does not have a serious problem of common method bias.

## 3 Result

### 3.1 Descriptive statistics analysis

In total, 10,273 (5,352 were male, 4,921 were females) usable responses were obtained. The average age of the participants was 14.96 years (SD = 1.86 years). Table 1 shows the demographic characteristics of the sample. In addition, the average scores of text intimidation, public texting, kicking out of group chats, spreading rumors, and verbally abusing others online reported by the participants in the last three months were 1.04, 1.05, 1.07, 1.04, and 1.06, respectively.

**Table 1:** Frequency statistics of demographic variables in the sample (*N* = 10,273).

Variables		Frequency	Percentage	*M*	*SD*
Sex	Male	5,352	52.10%	0.48	0.50
	Female	4,921	47.90%		
Grade	7 grade	1972	19.20%	3.39	1.71
	8 grade	1800	17.50%		
	9 grade	1,378	13.40%		
	10 grade	1896	18.50%		
	11 grade	1823	17.70%		
	12 grade	1,404	13.70%		
Family income/monthly	Less than 1,000 Yuan	1,032	10.00%	3.99	1.70
	1,000 Yuan – Less than 2000 Yuan	1,359	13.20%		
	2000 Yuan – Less than 3,000 Yuan	1708	16.60%		
	3,000 Yuan – Less than 4,000 Yuan	1803	17.60%		
	4,000 Yuan – Less than 5,000 Yuan	1,368	13.30%		
	5,001 Yuan and above	3,003	29.20%		
Household registration	Rural	3,577	34.80%	0.65	0.48
	Urban	6,696	65.20%		
Only child	Yes	7,903	76.90%	0.23	0.42
	No	2,370	23.10%		
Left-behind child	Yes	3,583	65.10%	0.35	0.48
	No	6,690	34.90%		
Parental divorce	Yes	725	92.90%	0.07	0.26
	No	9,548	7.10%		

“Less than” in this table does not include the number, “and above” includes the number.

### 3.2 Correlation analyses

Correlation analyses were conducted for cyberbullying, domestic violence exposure, depression, and self-control, and the correlation matrix for each variable was shown in Table 2.

**Table 2:** Pearson’s correlation between variables.

Variable	1	2	3	4
1.Cyberbullying behavior	1			
2.Domestic violence exposure	0.266^***^	1		
3.Depression	0.131^***^	0.310^***^	1	
4.Self-Control	−0.087^***^	−0.146^***^	−0.405^***^	1

^***^p < 0.001.

The results showed that cyberbullying behavior was positively correlated with domestic violence exposure and depression and negatively correlated with self-control; violence exposure was positively correlated with depression and negatively correlated with self-control, and depression was negatively correlated with self-control.

### 3.3 Moderated mediation effects test

First, by controlling the seven demographic variables, namely gender, grade, family income, family residency, only child status, left-behind children status, and parent’s divorce status, Model 59 was used to examine the direct effects of domestic violence exposure on cyberbullying, the mediating role of depression between domestic violence exposure and cyberbullying, and the moderating role of self-control on both the direct and indirect pathways, where the scores substituted into the model for the calculations were the average scores for each variable.

Results of bias-corrected thousandths Bootstrap Analyses showed (Table 3) that domestic violence exposure scores were positively associated with cyberbullying scores (*β* = 0.20, *p* < 0.001), **H1 was supported**; there was a positive association between domestic violence exposure exposure scores and depressed mood scores (*β* = 0.25, *p* < 0.001) and a positive association between depressed mood scores and cyberbullying scores (*β* = 0.06, *p* < 0.001), thus, depression partially mediated the relationship between domestic violence exposure and cyberbullying scores and **H2 was supported**; The coefficient of the interaction term between domestic violence exposure scores and self-control scores was negatively associated with cyberbullying scores (*β* = −0.09, *p* < 0.001), suggesting that self-control buffers the effects of domestic violence exposure on cyberbullying, and **H3a was supported**; The relationship between the coefficient of the interaction term between domestic violence exposure scores and self-control scores and depression scores was not significant, and **H3b was not supported**; There was a positive association between the coefficient of the interaction term between the depression score and the self-control score and the cyberbullying score (*β* = 0.03, *p* < 0.001), suggesting that self-control intensified the effect of depression on cyberbullying, **H3c was rejected**.

**Table 3:** Result of moderated mediation model test.

Variable	Outcome variable: depression			Outcome variable: cyberbullying		
	*β* value	*t* value	95% CI	*β* value	*t* value	95% CI
Sex	−0.27^***^	−15.58	−0.31 ~ −0.24	0.13^***^	6.94	0.10 ~ 0.17
Grade	0.01	0.01	−0.01 ~ 0.01	0.01	1.50	−0.01 ~ 0.02
Family income	−0.01	−1.07	−0.02 ~ −0.01	−0.01	−0.54	−0.02 ~ 0.01
Household registration	−0.01	−0.46	−0.05 ~ 0.03	0.01	0.60	−0.03 ~ 0.06
Only child status	0.04	1.55	−0.01 ~ 0.08	0.01	0.55	−0.04 ~ 0.06
Left-behind child status	0.03	1.58	−0.01-0.07	0.01	0.18	−0.04 ~ 0.05
Parental marital status	0.12^**^	3.38	0.05 ~ 0.19	0.01	0.25	−0.06 ~ 0.08
Family violence exposure	0.25^***^	23.36	0.23 ~ 0.27	0.20^***^	16.74	0.18 ~ 0.23
Self-control	−0.34^***^	−37.81	−0.36 ~ −0.32	−0.04^**^	−3.41	−0.06 ~ −0.02
Domestic violence exposure*self-control	0.01	0.17	−0.02 ~ 0.02	−0.09^***^	−6.54	−0.11 ~ −0.06
Depression				0.06^***^	5.01	0.04 ~ 0.08
Depression*self-control				0.03^**^	2.68	0.01 ~ 0.05
*R* ^Square^	0.22			0.08		
*F*	293.28^***^			76.80^***^		

^*^p < 0.05. ^**^p < 0.01.^***^p < 0.001.

**Figure 2:**
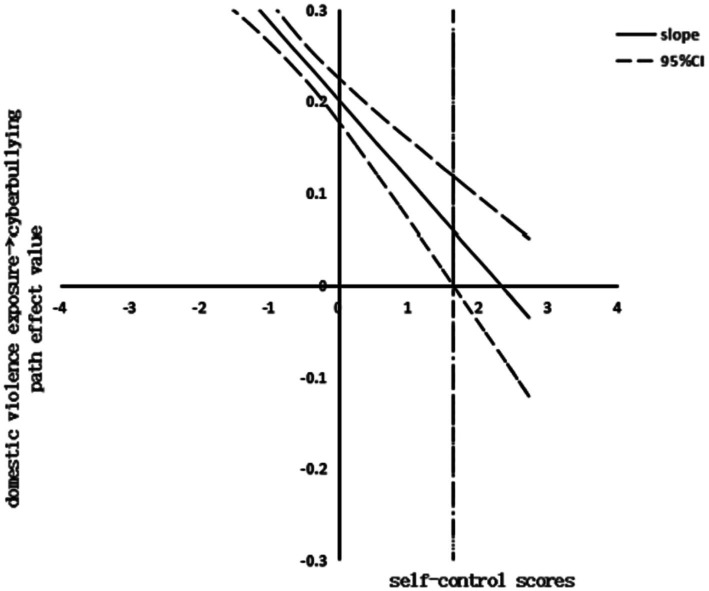
The moderating effect of self-control between domestic violence exposure and cyberbullying.

**Figure 3:**
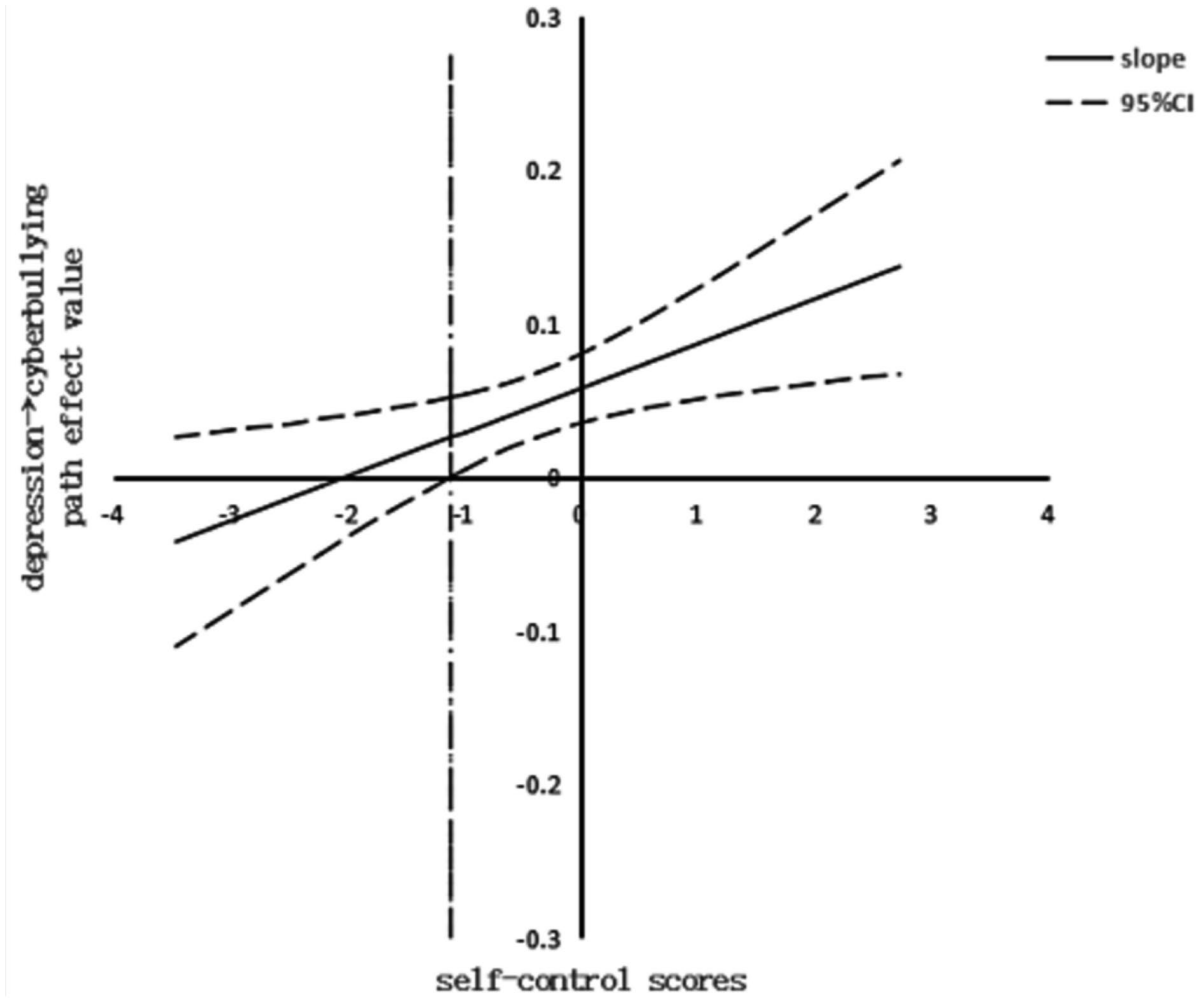
The moderating effect of self-control between depression and cyberbullying.

Finally, the visualization of the moderating effect of the J-N method was conducted. The effect plot of self-control moderating the relationship between exposure to domestic violence and cyberbullying is shown in Figure 2. When the value of self-control was taken to be less than 1.64, the confidence interval did not contain 0. The moderating effect was statistically significant, and the positive correlation between secondary school students’ perceived level of exposure to domestic violence and cyberbullying weakened with the increase in the level of self-control. The effect plot of self-control moderating the relationship between depression and cyberbullying is shown in Figure 3. When the value of self-control was taken to be greater than −1.11, the confidence interval did not contain 0. The moderating effect was statistically significant. The positive correlation between the level of depression and cyberbullying among secondary school students increased with the level of self-control.

## 4 Discussion

Previous studies on domestic violence exposure and bullying have focused on physical bullying behavior (37, 38), yet cyberbullying, as an extension of bullying phenomena in the online world, also deserves special attention. This study completes the expansion of the established empirical findings by exploring the relation of domestic violence exposure and cyberbullying behavior, the mediating effect of depression between domestic violence exposure and cyberbullying, and the moderating effect of self-control.

This study found that domestic violence exposure is positively associated with cyberbullying behavior among secondary school students. The results are consistent with the findings of previous studies (10–12) and support the idea of social learning theory. Violence in a family setting usually manifests in physical conflict between intimate partners or physical abuse to the adolescents themselves. It provides adolescents with behavioral patterns to observe and learn from. Adolescents who are repeatedly exposed to violent situations subconsciously develop learning and internalization of aggressive knowledge structures by observing the behavior of role models during violent events, which activates and reinforces the individual’s aggression schema and will increase the likelihood of using violence in real life and online life. Ultimately, the individual acquires aggressive beliefs and patterns of aggressive behavior and gradually develops aggressive personality traits.

Stimulating violent messages at home also leads to the rupture of the parent–child bond, weakening the individual’s internal desire to suppress anger and aggression. In real life, attacking others is considered immoral, and the Internet provides a “cover” for this with its nature of being anonymous and exceeding the limitations of time and space. As a result, adolescents growing up during the Internet age are more likely to use cyberbullying in the virtual space to resolve interpersonal conflicts. The results suggest that family members should manage their own emotions and behaviors well to avoid all kinds of psychological and physical harm caused by violent attitudes and behaviors to their children.

The test of depression’s mediating role indicated that it partially mediated the relationship between domestic violence exposure and cyberbullying among secondary school students. Domestic violence exposure can either directly affect cyberbullying among secondary school students or indirectly affect cyberbullying among secondary school students through depression. At the same time, this finding validates the general strain theory that stress reinforces aggressive behavior in individual adolescents by increasing negative emotional experiences (e.g., depression) (39).

Specifically, exposure to long-term violence at home creates negative stimuli for adolescents. Adolescents are exposed to such “harmful” stressful situations and cannot legitimately disengage from them in the same way as adults are. They are susceptible to developing depressive symptoms such as distress, hopelessness, and negative perceptions of self and the world, which in turn reduces the ability to regulate emotions and can lead to cyberbullying.

The study found that self-control significantly moderated the relationship between domestic violence exposure and cyberbullying among secondary school students. This predictive effect was stronger at low levels of self-control. This finding supports the dual-systems model that suggests violent cues witnessed and experienced by secondary school students at home serve as accelerating “catalysts” for cyberbullying behavior. The self-control system enhances or weakens this catalytic process. The results suggest that parents and teachers should pay attention to their children’s emotion condition and channel negative emotions in time to avoid serious psychological and behavioral problems.

The self-control system, on the other hand, enhances or weakly regulates this catalytic process (40) secondary school students with high self-control will fully utilize their cognitive resources to assess the appropriateness and risk of cyberbullying behavior during the process of using the Internet. His internalized moral beliefs, social customs, and other reflective systems will make rational assessments, and even if they are repeatedly exposed to violent cues, they will limit their aggressive impulses. In other words, high levels of self-control alleviate the catalytic effect of domestic violence exposure on cyberbullying.

On the contrary, when secondary school students with low levels of self-control use the Internet, their cognitive resources are under-called, their egos are more depleted, they tend to seek stimulation and risky behaviors without considering the long-term consequences of their behaviors, and they are susceptible to cognitive impulses. Instead, they are unable to assess risks rationally, so they are less likely to consider more profound moral beliefs and tend to fail in self-control, which can lead to committing bullying behaviors in cyberspace, thus exacerbating the catalytic effect of exposure to domestic violence on cyberbullying among secondary school students.

However, the moderating effect of self-control on the relationship between domestic violence exposure and depression among secondary school students is not significant. On the one hand, it may be due to the strong and stable psychological trauma caused by witnessing or experiencing domestic violence stimuli (41). On the other hand, the self-control of middle school students is not complete enough to develop the power to resist the generation of depression caused by domestic violence (42), and the strong impact of domestic violence exposure and the weak self-control resulted in the moderating effect failing to show statistical significance.

It is worth noting that the moderating effect of self-control on the relationship between depression and cyberbullying among secondary school students was contrary to the hypothesis, i.e., self-control would exacerbate the effect of depression on cyberbullying. This effect was not difficult to understand in the context of Chinese culture, which is traditionally based on Confucianism and has developed a strong introverted national character. Adolescents with a high level of self-control are more inclined to suppress the expression of negative emotions (43), and are more likely to accumulate large amounts of depression. When depression reached the threshold, secondary school students who were under great pressure to go to higher education were more likely to develop aggressive behaviors if they did not receive effective social support (prolonged exposure to domestic violence stimuli weakens perceptions of family support) (44). The anonymous environment of the Internet contained a disinhibition effect, which made adolescents tend to show high moral excuses and low guilt, and it was easier for them to distort the consequences of their own immoral behaviors and adjust the attribution to excuse and defend themselves. Therefore, it could be a coping strategy for adolescents with a high level of self-control to resolve their depression. The results suggest focusing on different mechanisms of self-control in order to better prevent cyberbullying behaviors among secondary school students.

## 5 Research limitation

This study has several limitations. First, the self-report method used in this study to measure cyberbullying may have underestimated the occurrence of bullying, and future research could integrate a variety of measures such as peer, parent, or teacher ratings. Second, given the cross-sectional nature of the study, we were able to capture the influence of self-control on the contemporaneous effects of their regulation. However, the long-term effects of perceived domestic violence on cyberbullying are still unknown, and future research could examine the study results using a longitudinal follow-up design. Finally, due to the limited length of the questionnaire, we were not able to cover other vital characteristics of the respondents, such as other psychological and physiological differences among individuals, and we may not have been able to observe other confounders affecting cyberbullying. Future studies could be more well-developed by adding more information or in-depth interviews.

## 6 Conclusion

This study obtained the following conclusions:

1. Domestic violence exposure has a significant positive associated with cyberbullying behavior among secondary school students, i.e., the higher the perceived domestic violence exposure of secondary school students, the more likely cyberbullying is to occur;
2. The mediating effect of depression partially exists between domestic violence exposure and cyberbullying behavior among secondary school students, i.e., the higher the perceived domestic violence exposure of secondary school students, the stronger the depressed mood, and the more likely cyberbullying would occur; and
3. Self-control alleviated the effects of domestic violence exposure on cyberbullying among secondary school students but intensified the effects of depression on cyberbullying among secondary school students.

## Data availability statement

The raw data supporting the conclusions of this article will be made available by the authors, without undue reservation.

## Author contributions

DC: Writing – original draft. BX: Writing – review & editing. JC: Writing – review & editing.
